# Which patient-specific parameters correlate with operation time for total hip arthroplasty? — A retrospective analysis of the direct anterior approach

**DOI:** 10.1007/s00264-023-05841-7

**Published:** 2023-06-03

**Authors:** Sebastian von Hertzberg-Boelch, Laura Mueller, Ioannis Stratos, Joerg Arnholdt, Boris Holzapfel, Maximilian Rudert

**Affiliations:** 1 Department of Orthopaedic Surgery, Julius-Maximilian University, Würzburg, Germany; 2 LVR Klinik für Orthopädie Viersen, Viersen, Germany; 3 Department of Orthopaedics and Trauma Surgery, Musculoskeletal University Center Munich (MUM), University Hospital, Munich, Germany

**Keywords:** DAA, Anterior approach, Total Hip Arthroplasty, Cementless THA, Obesity, BMI

## Abstract

**Purpose:**

The current study aims to identify patient-specific factors that correlate with operation time for total hip arthroplasty (THA) performed via the direct anterior approach (DAA).

**Methods:**

In this retrospective study, patient-specific factors were tabulated from the charts and measured from preoperative templating radiographs. These factors were correlated with operation time by bivariate analysis. Significant factors were used for stepwise multiple regression analysis.

**Results:**

Nine hundred-sixty procedures were included. BMI (*R* = 0.283), the distance from the superior iliac spine to the greater trochanter (DAA Plane) (R = − 0.154), patients age (R = 0.152) and the abdominal fat flap (R = 0.134) showed the strongest correlations (*p* < 0.005) with operation time. The multiple regression model including BMI, Kellgren and Lawrence Score, Age, DAA Plane and the Canal to Calcar ratio had the best predictive accuracy (corrected *R*^2^ = 0.122).

**Conclusions:**

Patient-specific factors that make the entry into the femur difficult correlate significantly with operation time of THA via the DAA.

## Introduction

Total hip arthroplasty (THA) has been recognized as one of the most significant surgical advances of the last century [1], and its use is increasingly common worldwide. In Germany, for example, THA rates are expected to increase by over 60% in the next 40 years [2]. THA has now become a highly standardized procedure, and THA surgeons are now considering operation time from both medical and economic perspectives.

Operation room planning is currently based on allocating standard times to a specific procedure, such as cementless primary THA. These standard times are derived from the average in-house operation time of this specific procedure. However, this approach to scheduling THA procedures is sensitive for deviations [3]. When procedures are completed faster than anticipated, resources remain unused, and when they take longer than expected, this can lead to congestion. As a result, cost analysis of THA has shown that the largest portion of direct cost variation is due to the time taken for the surgery [4].

Furthermore, operation time significantly affects the outcome after joint replacement. Increased operation time correlates with readmission rates [5] and is a significant factor for short and long-term complications, in particular periprosthetic infection [6]. Different approaches for THA have been compared in terms of complication rates and operation time [7]. From time to time the direct anterior approach (DAA) is criticized for potentially resulting in higher rates of infection and femoral complications compared to other approaches [8] and some have suggested that it is only appropriate for certain patients [9]. Nevertheless, the DAA is becoming increasingly popular worldwide because it is the sole approach to the hip joint that passes through an intermuscular and internervous plane [10], leading to a short recovery period [11]. However, the authors are not aware of a study that has investigated patient specific factors, which affect operation time for THA performed with the DAA.

Therefore, the current study aims to identify patient-specific factors that correlate with operation time for THA performed via the DAA.

## Materials and methods

Approval for this retrospective study was waived by the institution’s ethics committee. All THAs performed at the study institution via the DAA in patients without additional private insurance and with the hospital’s standard implants between 2015 and 2018 were included. Implantations performed by surgeons with fewer than 50 THA implantations or revisions at the hip per annum were excluded.

### THA implantation

At the study institution more than 700 THAs are performed per year via the DAA in supine position. The technique through Hueter’s interval is described in detail elsewhere [12]. A proximally plasma sprayed monoblock stem with cementless metaphyseal tapered wedge fixation (ML Taper FA. ZimmerBiomed) and a hemispherical but flattened cup with a rough-blasted surface (Allofit Alloclassic S Fa. ZimmerBiomed) are used as implants. Intraoperative fluoroscopy is used to verify correct implant placement before wound closure. Used implant sizes were tabulated from the digital patient charts.

### Patient-specific factors

Patient-specific factors were tabulated from the digital patient charts. Preoperative templating radiographs (anterior to posterior radiographs of the pelvis; centre beam on the symphysis, the patient in supine position, and about 20° of internal rotation of both thighs) were used to characterize the patients individual anatomy. Only radiographs with sufficient quality (symmetric obturator foramen and centred position of the sacrum, complete depiction of both proximal femora) were used. Measurements were performed with a THA planning software after calibration (mediCAD Fa. medicad Hectec Germany). The hips were classified as coxa vara, norma, or valga if the tangent to the tip of the greater trochanter, which was orthogonal to the femoral shaft axis, ran through the lower, the middle, or the upper third of the femoral head. The hips were classified as coxa profunda or protrusio depending on whether the femoral head touched or protruded the Kohler’s line. Arthrosis was classified according to Kellgren and Lawrence (KLS) [13]. If present, dysplasia was classified according to Hartofilakidis [14]. The femur was classified according to Dorr and evaluated according to the cortical thickness index (CTI) as well the canal to calcar ratio (CCR) [15]. The femoral head extrusion index [16], the centre to edge angle according to Wiberg [17], the critical trochanteric angle [18], and the age of the patient at operation were calculated. The distance from the anterior superior iliac spine (ASIS) to the ipsilateral greater trochanter was measured in mm to quantify the plane for the approach to the hip joint with the DAA (DAA plane (DAAP)). The contour of the abdominal fat flap (AFF) was described as not present (0), as running above the acetabulum (I), through the femoral head (II), across the femoral neck (III), across the intertrochanteric region (IV), or below the lesser trochanter (V).

### Statistics

Patient characteristics are given as mean and range. Patient-specific factors are given as mean and standard deviation. To evaluate the patient-specific factors that correlate with operation time, a bivariate analysis was conducted first. Bivariate correlation of operation time with metric parameters was evaluated according to Pearson, ordinal and nominal scaled parameters were correlated with the Spearman-Rho coefficient (*R*). To minimize collinearity, patient-specific factors were also evaluated for bivariate correlation. For collinearity, a correlation < 0.8 was considered irrelevant [19]. Significant factors from bivariate analysis were used for a stepwise multiple regression analysis. Only complete data set was included. The resulting multiple regression models were evaluated for significance with an ANOVA and predictive accuracy of the model was described with the corrected *R*^2^. Significancy was set at *p* < 0.05. Statistics were performed with SPSS Statistics 28.

## Results

A total of 1223 THA procedures matched the inclusion criteria. However, 263 procedures were performed by surgeons with less than 50 THA procedures per annum and therefore were excluded from the study, leaving 960 THA implantations for analysis. These were 470 men and 490 women, the average age was 64.3 years (22–90), the average BMI was 28.6 kg/cm^2^ (17.2–59.5), and the average ASA score 2 (1–4). 497 (51.8%) procedures were performed on the left hip and the remaining 463 (48.2%) on the right. Median stem and cup sizes used were 11 (4–20) and 52 (44–62), respectively.

The 90-day complication rate with revision was 3.3%. These were 17 wound-healing problems requiring revision, three early infections, six dislocations, four fractures around the stem, and one early aseptic loosening.

Mean operation time was 59 min (17.49). The two parameters with the strongest correlation to operation time in the bivariate analysis were the BMI (*R* = 0.283) followed by the DAAP (*R* =  − 0.154) (Tables 1 and 2).

**Table 1:** Metric patient-specific parameters with correlation coefficient (*R*) and significance (*p*) from bivariate analysis

Parameter	Mean (SD)	*R*	*p*
BMI kg/cm^2^	28.56 (5.18)	0.283	1.5802E-18*
DAA plane (mm)	65.1 (11.92)	− 0.154	0.000015*
Age	64.3 (10.75)	0.152	0.000002*
Calcar to canal ratio	0.63 (0.096)	0.137	0.000023*
Flexion during range of motion test (degrees)	89 (14)	− 0.129	0.000097*
Cortical thickness index	0.59 (0.067)	− 0.024	0.465077
Centre to edge angle (degrees)	40.43 (12.26)	0.013	0.690162
Critical trochanteric angle (degrees)	18.15 (8.96)	0.007	0.836574

*BMI*, body mass index; *DAA*, direct anterior approach; *SD*, standard deviation

*, significant

**Table 2:** Patient-specific parameters sorted by classification with prevalence and the correlation coefficient (*R*) and significance (*p*) from bivariate analysis

Parameter (classification)	Prevalence N (%)	*R*	*p*
AFF (0/I/II/III/IV/V)	608 (63.3) / 116 (12.1) / 103 (10.7) / 65 (6.8) / 42 (4.4) / 26 (2.7)	0.138	0.000018*
KLS (1 to 4)	2 (0.2) / 128 (113.3) / 605 (63.0) / 225 (23.4)	0.118	0.000253*
Coxa profunda/protrusio	175 (18.2) / 49 (5.1)	0.101	0.001847*
DORR type 1/2/3	729 (75.9) / 198 (20.6) / 33 (3.4)	0.084	0.009178*
Sex (women/men)	490 (51.0)/470 (49.0)	-0.074	0.021092*
ASA Score (1/2/3/4)	67 (7.0)/640 (66.6)/ 238 (24.8)/ 3(0.3)	0.064	0.084957
Hartofilakidis (I/IIA/IIB)	207 (21.6) / 67 (7.0) / 4 (0.4)	-0.036	0.272647
Coxa norma/valga/vara	570 (59.4) / 105 (10.9) / 275 (28.6)	-0.008	0.806016

*AFF*, abdominal fat flap; *KLS*, Kellgren and Lawrence score; *ASA*, American Society of Anesthesiologists﻿

*, significant

The combination of the following covariates resulted in the stepwise multiple linear regression model (*N* = 752) with the highest corrected *R*^2^: BMI, KLS, Age, CCR and DAAP (Table 3).

**Table 3:** Models from stepwise multiple regression analysis with corrected correlation (*R*^2^) and level of significance (*p*)

Model	Included covariates	Corrected *R*^2^	*p*
1	BMI	0.075	1.38E-14*
2	BMI, KLS	0.093	4.3901E-17*
3	BMI, KLS, Age	0.112	1.0658E-19*
4	BMI, KLS, Age, CCR	0.118	2.7643E-20*
5	BMI, KLS, Age, CCR, DAAP	0.122	1.6558E-20*

*BMI*, body mass index; *KLS*, Kellgren and Lawrence Score; *DAAP*, Direct Anterior Approach Plane; *CCR*, Calcar to Canal Ratio

*, significant

## Discussion

Despite the high standardization of the procedure in high-volume arthroplasty centres, THA implantation takes longer in some patients than in others. Prolonged operation time is associated with a higher risk of complications [5] and can lead to the wastage of resources [3]. However, it remains unclear which factors are linked to longer operation time. Therefore, the objective of the current study was to examine patient-specific factors that are correlated with extended operation time during THA.

The BMI was found to have the strongest correlation with operation time. Increased operation time has been reported for obese patients before. For the minimally invasive anterolateral approach with short stems, for instance, patients with a BMI above 30 kg/cm^2^ had a significant longer operation time compared to those with a BMI below 24 kg/cm^2^ [20]. It is well established that obesity can lead to longer operation time for THA [21], and as the results show, this is also true for THA via the DAA. Obesity makes THA more difficult by reducing the “working space” and obscuring the landmarks due to the presence of fat around the hip joint [22]. As a result, modifications to the DAA such as the “Bikini incision” have been proposed for obese patients [23].

The current study found that next to the BMI additional parameters which quantify the working space are significant covariates for operation time via the DAA. When the access through Hueter’s interval is narrow, it can be challenging to get “around the corner” to the femur [22]. Then, the mobilization of the femur by a stepwise release technique is key to the DAA [24]. The current results confirm the surgeon’s experience, that particularly parameters that hinder positioning of the femur for broaching correlate with longer operation time: Younger patients can have stronger tissue, whereas advanced osteoarthritis can impede mobilization of the femur by capsular contracture or hypertrophie. An overhanging abdominal fat flap can hamper untroubled use of the broaching instruments. A small DAAP may afford more capsular releases to open the corridor the femur. The difficulty to gain optimal entry explains why a higher rate of early femoral failures compared to other approaches has been described [25]. Additionally, this difficulty has led to various techniques for the DAA, such as lateral decubitus positioning [26], the use of specific retractor holding devices [27] or the use of a traction table [28]. Other authors even describe extensive release algorithms involving the obturator externus muscle [29].

Hence, the large number of patients in the current study, the limited predictive accuracy is certainly caused by the plethora of variables that in the end additionally influence the operation time of THA. Training of interns, nurses and students or hampering problems with the instruments cannot be routinely documented and are not patient-specific.

The correlation of operation time with the performing surgeon was minimized by excluding surgeons in the learning curve for THA via the DAA. However, the current regression model is based on six different surgeons. Among these surgeons predictive accuracy for the derived regression model (corrected *R*^2^) ranged from 0.049 to 0.219. When applied to each individual surgeon, the described regression model was significant (*p* < 0.05) in four. Interestingly, this observation is not explained by the different levels of experience of these surgeons: While a comparatively junior surgeon had an average operation time of 53.9 (19.6), a senior surgeon with far more experience had an average operation time of 65.3 (16.0) min. The regression model was significant for both of these surgeons and additionally for the two most experienced ones. Although the current study aimed to identify patient-specific factors, the corrected *R*^2^ for multiple regression model including the surgeon as a covariate, was 0.134 and *p* < 0.001.

The few other studies that have explored predictors of operation time for joint arthroplasty yielded a higher predictive accuracy. Wu et al. developed a prediction model for operation time of THA revision. Their predictors were additional surgical steps, for instance, whether or not a fixed stem or a remaining cement mantel had to be removed [30]. Machine learning is a promising approach for predicting operation time, as larger datasets can be processed to improve predictive accuracy. Yeo et al. reported excellent results for a predictive model for total knee arthroplasty, but their predictors were variables that influenced the implantation concept [31]. In contrast, the current study focuses on patient-specific predictors for operation time within one consistent treatment concept.

It is clear that the technical implementation of the standardized steps of the operation is subject to the patient’s individual anatomy. It is also clear that experienced surgeons are able to identify patients that require more operation time. However, the current study points out that factors that tighten the DAA working spacer significantly correlate with longer operation time of THA via the DAA.

## Data Availability

All data is available from the corresponding author upon reasonable request.

## References

[1] Learmonth ID, Young C, Rorabeck C (2007). The operation of the century: total hip replacement. Lancet.

[2] Klug A, Pfluger DH, Gramlich Y, Hoffmann R, Drees P, Kutzner KP (2021). Future burden of primary and revision hip arthroplasty in Germany: a socio-economic challenge. Arch Orthop Trauma Surg.

[3] Yeo I, Klemt C, Melnic CM, Pattavina MH, De Oliveira BMC, Kwon YM (2022). Predicting surgical operative time in primary total knee arthroplasty utilizing machine learning models. Arch Orthop Trauma Surg.

[4] Rudy MD, Bentley J, Ahuja N, Rohatgi N (2020). Determinants of cost variation in total hip and knee zarthroplasty: implications for alternative payment models. J Am Acad Orthop Surg.

[5] Surace P, Sultan AA, George J, Samuel LT, Khlopas A, Molloy RM (2019). The association between operative time and short-term complications in total hip arthroplasty: an analysis of 89,802 Surgeries. J Arthroplasty.

[6] Duchman KR, Pugely AJ, Martin CT, Gao Y, Bedard NA, Callaghan JJ (2017). Operative time affects short-term complications in total joint arthroplasty. J Arthroplasty.

[7] Kraus KR, Dilley JE, Ziemba-Davis M, Meneghini RM (2022). Procedure duration, time under anesthesia, and readmissions in direct anterior and posterior approach total hip arthroplasty. J Arthroplasty.

[8] Huang XT, Liu DG, Jia B, Xu YX (2021). Comparisons between direct anterior approach and lateral approach for primary total hip arthroplasty in postoperative orthopaedic complications: a systematic review and meta-analysis. Orthop Surg.

[9] Antoniadis A, Dimitriou D, Flury A, Wiedmer G, Hasler J, Helmy N (2018). Is direct anterior approach a credible option for severely obese patients undergoing total hip arthroplasty? A matched-control, retrospective, clinical study. J Arthroplasty.

[10] Stratos I, Heller KD, Rudert M (2021). German surgeons' technical preferences for performing total hip arthroplasties: a survey from the National Endoprosthesis Society. Int Orthop.

[11] Goebel S, Steinert AF, Schillinger J, Eulert J, Broscheit J, Rudert M (2012). Reduced postoperative pain in total hip arthroplasty after minimal-invasive anterior approach. Int Orthop.

[12] Rudert M (2019) Zementfreie Hüftendoprothese: minimalinvasiver anteriorer Zugang. Berlin Heidelberg Springer. 10.1007/978-3-662-59891-7

[13] Kohn MD, Sassoon AA, Fernando ND (2016). Classifications in brief: Kellgren-Lawrence classification of osteoarthritis. Clin Orthop Relat Res.

[14] Holzapfel BM, Burklein D, Greimel F, Noth U, Hoberg M, Gollwitzer H (2011). Total hip replacement in developmental dysplasia: anatomical features and technical pitfalls. Orthopade.

[15] Nash W, Harris A (2014). The Dorr type and cortical thickness index of the proximal femur for predicting peri-operative complications during hemiarthroplasty. J Orthop Surg.

[16] Murphy SB, Ganz R, Muller ME (1995). The prognosis in untreated dysplasia of the hip. A study of radiographic factors that predict the outcome. J Bone Joint Surg.

[17] Wiberg G (1939) Studies on dysplastic acetabula and congenital subluxation of 320 the hip joint: with special reference to the complication ofosteoarthritis. Acta Chirurgica

[18] Haversath M, Busch A, Jager M, Tassemeier T, Brandenburger D, Serong S (2019). The 'critical trochanter angle': a predictor for stem alignment in total hip arthroplasty. J Orthop Surg Res.

[19] Field AP (2018) Discovering statistics using IBM SPSS statistics. In: Oaks T, editor. London. p. 402

[20] Luger M, Hochgatterer R, Schopper C, Pisecky L, Allerstorfer J, Klasan A (2021). Obesity in short stem total hip arthroplasty using a minimally invasive supine anterolateral approach-a risk factor for short-term complications?. Int Orthop.

[21] Hartford JM, Graw BP, Frosch DL (2020). Perioperative complications stratified by body mass index for the direct anterior approach to total hip arthroplasty. J Arthroplasty.

[22] Holzapfel BM, Rak D, Kreuzer S, Arnholdt J, Thaler M, Rudert M (2021). Short stem hip arthroplasty via the minimally invasive direct anterior approach. Oper Orthop Traumatol.

[23] Corten K, Holzapfel BM (2021). Direct anterior approach for total hip arthroplasty using the "bikini incision". Oper Orthop Traumatol.

[24] Nistor DV, Caterev S, Bolboaca SD, Cosma D, Lucaciu DOG, Todor A (2017). Transitioning to the direct anterior approach in total hip arthroplasty. Is it a true muscle sparing approach when performed by a low volume hip replacement surgeon?. Int Orthop.

[25] Meneghini RM, Elston AS, Chen AF, Kheir MM, Fehring TK, Springer BD (2017). Direct anterior approach: risk factor for early femoral failure of cementless total hip arthroplasty: a multicenter study. J Bone Joint Surg Am.

[26] Camenzind RS, Stoffel K, Lash NJ, Beck M (2018). Direct anterior approach to the hip joint in the lateral decubitus position for joint replacement. Oper Orthop Traumatol.

[27] Morgenstern R, Su EP (2018). The gripper table mounted retraction system: a tireless surgical assistant. Semin Arthroplast.

[28] Goldberg TD, Kreuzer S, Randelli F, Macheras GA (2021). Direct anterior approach total hip arthroplasty with an orthopedic traction table. Oper Orthop Traumatol.

[29] Chughtai M, Samuel LT, Acuna AJ, Kamath AF (2019). Algorithmic soft tissue femoral release in anterior approach total hip arthroplasty. Arthroplast Today.

[30] Wu A, Weaver MJ, Heng MM, Urman RD (2017). Predictive model of surgical time for revision total hip arthroplasty. J Arthroplasty.

[31] Tuwatananurak JP, Zadeh S, Xu X, Vacanti JA, Fulton WR, Ehrenfeld JM (2019). Machine learning can improve estimation of surgical case duration: a pilot study. Jorunal Med Syst.

